# Comparison of Beta-value and M-value methods for quantifying methylation levels by microarray analysis

**DOI:** 10.1186/1471-2105-11-587

**Published:** 2010-11-30

**Authors:** Pan Du, Xiao Zhang, Chiang-Ching Huang, Nadereh Jafari, Warren A Kibbe, Lifang Hou, Simon M Lin

**Affiliations:** 1 Northwestern University Biomedical Informatics Center (NUBIC), NUCATS, Feinberg School of Medicine, Northwestern University, Chicago, IL 60611, USA; 2 Department of Preventive Medicine, Feinberg School of Medicine, Northwestern University, Chicago, IL 60611, USA; 3 The Robert H. Lurie Comprehensive Cancer Center, Northwestern University, Chicago, IL 60611, USA; 4 Center for Genetic Medicine, Feinberg School of Medicine, Northwestern University, Chicago, IL 60611, USA

## Abstract

**Background:**

High-throughput profiling of DNA methylation status of CpG islands is crucial to understand the epigenetic regulation of genes. The microarray-based Infinium methylation assay by Illumina is one platform for low-cost high-throughput methylation profiling. Both Beta-value and M-value statistics have been used as metrics to measure methylation levels. However, there are no detailed studies of their relations and their strengths and limitations.

**Results:**

We demonstrate that the relationship between the Beta-value and M-value methods is a Logit transformation, and show that the Beta-value method has severe heteroscedasticity for highly methylated or unmethylated CpG sites. In order to evaluate the performance of the Beta-value and M-value methods for identifying differentially methylated CpG sites, we designed a methylation titration experiment. The evaluation results show that the M-value method provides much better performance in terms of Detection Rate (DR) and True Positive Rate (TPR) for both highly methylated and unmethylated CpG sites. Imposing a minimum threshold of difference can improve the performance of the M-value method but not the Beta-value method. We also provide guidance for how to select the threshold of methylation differences.

**Conclusions:**

The Beta-value has a more intuitive biological interpretation, but the M-value is more statistically valid for the differential analysis of methylation levels. Therefore, we recommend using the M-value method for conducting differential methylation analysis and including the Beta-value statistics when reporting the results to investigators.

## Background

Methylation of cytosine bases in DNA CpG islands is an important epigenetic regulation mechanism in the organ development, aging and different disease statuses [1]. Hypermethylation of CpG islands located in the promoter regions of tumor suppressor genes has been firmly established as one of the most common mechanisms for gene regulation in cancer [2,3]. Therefore, high-throughput profiling of DNA methylation status of CpG islands is crucial for forwarding our understanding of the influence of epigenomics [4-6]. Microarray-based Illumina Infinium methylation assay has been recently used in epigenomic studies [7-9] due to its high throughput, good accuracy, small sample requirement and relatively low cost [1].

To estimate the methylation status, the Illumina Infinium assay utilizes a pair of probes (a methylated probe and an unmethylated probe) to measure the intensities of the methylated and unmethylated alleles at the interrogated CpG site [10]. The methylation level is then estimated based on the measured intensities of this pair of probes. To date, two methods have been proposed to measure the methylation level. The first one is called Beta-value, ranging from 0 to 1, which has been widely used to measure the percentage of methylation. This is the method currently recommended by Illumina [11,12]. The second method is the log2 ratio of the intensities of methylated probe versus unmethylated probe [13]. We have referred to it as the M-value method because it has been widely used in the mRNA expression microarray analysis. Since both methods have their own strengths and limitations, understanding the performance characteristics of both measures is very important in providing the best methylation analysis. We found some studies that optimized clustering methylation data using the Beta-value [14] method; but a rigorous comparison of the two methods has not been done. For this reason, we designed a titration experiment to compare and evaluate these two methods. In the following sections, we will first define these two methods and derive the relationship between them. Then we will evaluate the performance of these two methods in detecting differentially methylated CpG sites.

## Results

### Definition of Beta-value and M-value

The Beta-value is the ratio of the methylated probe intensity and the overall intensity (sum of methylated and unmethylated probe intensities). Following the notation used by Illumina methylation assay [12], Beta-value for an i^th ^interrogated CpG site is defined as:

(1) $$Beta_{i}=\frac{\max(y_{i,methy},0)}{\max(y_{i,unmethy},0)+\max(y_{i,methy},0)+\alpha }$$

where *y_i,menty_* and *y_i,unmenty_* are the intensities measured by the i^th ^methylated and unmethylated probes, respectively. To avoid negative values after background adjustment, any negative values will be reset to 0. Illumina recommends adding a constant offset *α* (by default, *α* = 100) to the denominator to regularize Beta value when both methylated and unmethylated probe intensities are low. The Beta-value statistic results in a number between 0 and 1, or 0 and 100%. Under ideal conditions, a value of zero indicates that all copies of the CpG site in the sample were completely unmethylated (no methylated molecules were measured) and a value of one indicates that every copy of the site was methylated. If we assume the probe intensities are Gamma distributed, then the Beta-value follows a Beta distribution. For this reason, it has been named the Beta-value.

The M-value is calculated as the log2 ratio of the intensities of methylated probe versus unmethylated probe as shown in Equation 2:

(2) $$M_{i}=\log_{2}\left(\frac{\max(y_{i,methy},0)+\alpha }{\max(y_{i,unmethy},0)+\alpha }\right)$$

Here we slightly modified the definition given in [13] by adding an offset *α* (by default, *α* = 1) to the intensity values to prevent unexpected big changes due to small intensity estimation errors, since for very small intensity values (especially between 0 and 1), small changes of the methylated and unmethylated probe intensities can result in large changes in the M-value. A M-value close to 0 indicates a similar intensity between the methylated and unmethylated probes, which means the CpG site is about half-methylated, assuming that the intensity data has been properly normalized by Illumina GenomeStudio or some other external normalization algorithm. Positive M-values mean that more molecules are methylated than unmethylated, while negative M-values mean the opposite. The M-value has been widely used in expression microarray analysis, especially two-color microarray analysis. Therefore, many existing microarray statistical frameworks using an M-value method can also be applied to methylation data analysis.

### Relationship between Beta-value and M-value

For Illumina methylation data, typically more than 95% of interrogated CpG sites have intensities (*y*_*i,unmethy*_+*y*_*i,methy*_) larger than 1000 (our evaluation dataset had 99.8% interrogated CpG sites with intensities higher than 1000.). Therefore, the relatively small offset value (i.e., 100) in the denominator of Equation 1 has negligible effect on the Beta-value for most interrogated CpG sites. Similarly, the offset *α* in Equation 2 is also ignorable for most interrogated CpG sites. Based on this observation, the relationship between Beta-value and M-value can be derived by substitution using Equation 1 and 2 (with the offset ignored):

(3) $$Beta_{i}=\frac{2^{M_{i}}}{2^{M_{i}}+1};M_{i}=\log_{2}\left(\frac{Beta_{i}}{1-Beta_{i}}\right)$$

**Figure 1:**
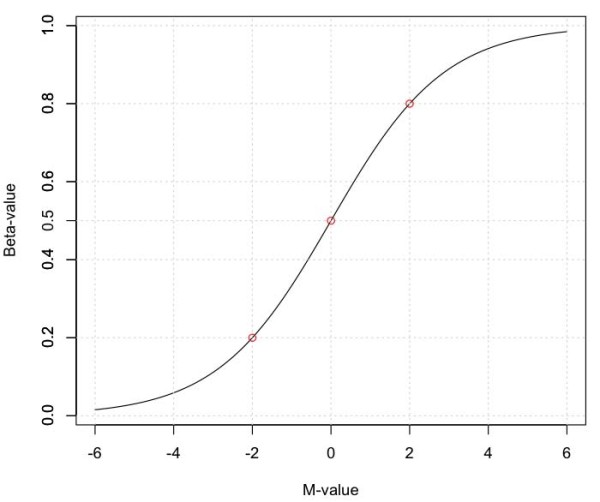
**The relationship curve between M-value and Beta-value**.

Equation 3 indicates that the relationship is a logistic function (shown as a base 2 logarithm instead of natural logarithm). Figure 1 shows the relationship curve between Beta and M-values. For example, Beta-values of 0.2, 0.5 and 0.8 correspond to M-values of -2, 0 and 2, respectively. An approximately linear relationship can be observed between Beta-value and M-value in the middle range (from 0.2 to 0.8 for Beta-values and from -2 to 2 for M-values). As shown in Figure 1, Beta-values are severely compressed at the extremes when compared with M-values. As shown in the following sections, the transformation of Beta-value into M-value provides a straightforward method for using the Beta-value statistic and obtaining the unique statistical properties of the M-value.

### Histograms of Beta-value and M-value

**Figure 2:**
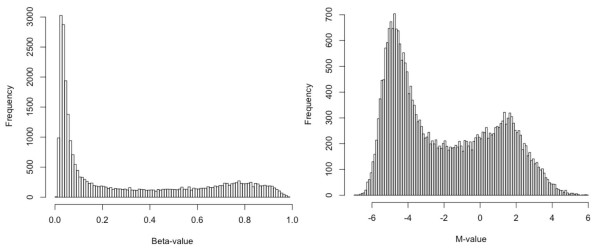
**The histograms of Beta-value (left) and M-value (right) (27578 interrogated CpG sites in total)**.

Figure 2 shows histograms of Beta-values and M-values for a typical sample measured by the Illumina Infinium HumanMethylation27 BeadChip, which interrogates 27,578 CpG sites in total, spread across promoter regions of 14,495 genes. The range of Beta-values is between 0 and 1, which can be interpreted as the approximation of the percentage of methylation for the population of a given CpG site in the sample. For M-values, it is difficult to directly infer the degree of methylation based on a single M-value, especially considering the range of M-values may change across different datasets. The histogram of M-values clearly shows a bimodal distribution, with one positive mode (methylated mode) and one negative mode (unmethylated mode). Conversely, because Beta-values are severely compressed in the low (between 0 and 0.2) and high (between 0.8 and 1) ranges compared with the M-value statistic, its bimodal distribution is less obvious. Therefore, the Beta-value has a direct correspondence with an intuitive mental model of methylation (% methylation for a given site) whereas the M-value may provide some insight into the distribution of methylation across the genome that is difficult to visualize with the Beta-value. See the Conclusions section for additional discussion of this point.

### The distribution of standard deviation across different methylation levels

**Figure 3:**
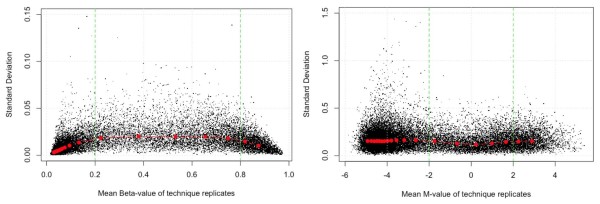
**The mean and standard deviation relations of technical replicates. Beta-value (left) and M-value (right)**.

In high-throughput statistical data analyses, many of them, like canonical linear models or ANOVA, assume the data is *homoscedastic*, i.e., the variable variances are approximately constant. The violation of this assumption, which is described as *heteroscedasticity *in statistics, imposes serious challenges when applying these analyses to high-throughput data [15]. A common way to check the *homoscedasticity *of the data is by visualizing the relations between mean and standard deviation [15,16]. Figure 3 shows the mean and standard deviation relations of the Beta-value and M-value, which were calculated based on technical replicates. The red dots represent the median standard deviation within a local window. The data was first ranked by mean methylation levels, and then binned into twenty non-overlapping windows, with each bin containing 5% of the data. The standard deviation of Beta-value is greatly compressed in the low (between 0 and 0.2) and high (between 0.8 and 1) ranges. This means Beta-value has significant *heteroscedasticity *in the low and high methylation range. The problem of *heteroscedasticity *is effectively resolved after transforming Beta-value to M-value using Equation 3. We can see M-value is approximately *homoscedastic*. Its standard deviation is approximately constant across the entire methylation range for M-values. The M-value statistic is therefore much more appropriate for the homoscedastic assumptions of most statistical models used for microarray analysis. It should be noted that other variance stabilization transformation methods may also be used to transform the Beta-value and stabilize the variance.

### Performance comparison between Beta and M-values

#### Evaluation dataset

Titration data has been widely used to evaluate the performance of new methods for analyzing mRNA expression microarrays [16,17]. To apply this practice to methylation analysis, we designed a methylation titration experiment that enables the evaluation of the performance of the Beta-value and M-value methylation analysis methods. Similar to the titration design using Goldengate methylation chips by Bibikova and et al. [12], we selected two samples known to contain significant methylation differences. Sample A is a B-lymphocyte sample from a male donor. Sample B is a colon cancer sample from a female donor. The sources of the methylation differences between sample A and B include: (1) gender differences; (2) pathological differences; (3) tissue differences. Samples A and B were mixed at five different titration ratios: 100:0, 90:10, 75:25, 50:50 and 0:100. The mixed samples were measured by Illumina Infinium HumanMethylation27 BeadChip with technical replicates. Please see the Methods section for a more detailed description.

As shown in Figure 1, the middle range of logistic transformation is approximately linear while the low and high ranges have clear nonlinear relationships between the Beta-value and M-value statistics. We have grouped the results of the transformations into three analysis groups, labeled as low, middle and high, with the middle analysis group corresponding to the approximately linear range and the low and high groups in the nonlinear range. This simplifies the analysis of the performance of each statistic.

Beta-value: low (0, 0.2), middle [0.2, 0.8] and high (0.8, 1).

M-value: low (-Inf, -2), middle [-2, 2] and high (2, Inf).

### Define differentially methylated CpG sites based on correlation

If an examined CpG site has a significant methylation difference between Sample A and B, its methylation profile should be correlated with the titration profile shown in Table 1. Therefore, we can use the correlation between the methylation and titration profile to validate whether the CpG site is differentially methylated between Sample A and B. Following similar criteria used in the expression titration microarray experiments [16,17], we claim a CpG site is differentially methylated between Sample A and B if its absolute correlation coefficients between titration and methylation profiles are larger than 0.8 (correlation p-value is about 0.05) both for Beta and M-value. There are 9845 investigated CpG sites satisfying this criterion. We treat them as True Positives (TP) to evaluate the performance of differential methylation analysis.

**Table 1:** Design of the methylation titration experiment

% mix of A and B for each sample	Mix1	Mix2	Mix3	Mix4	Mix5
**A**	100	90	75	50	0
**B**	0	10	25	50	100
N_tech_*	2	2	1	1	2

* N_tech _represents the number of technical replicates

### Performance comparison based on differential methylation analysis

One of the major statistical paradigms in expression microarray analysis has been the "Fold change-ranking with a non-stringent p-value cutoff" [18-20]. Under this framework, the CpG islands will be first subject to a low-stringency p-value threshold (p < 0.05 without the correction of multiple comparisons); and then ranked by fold changes. We hypothesized that M-value outperforms Beta-value under this statistical framework because M-value is more homoscedastic and therefore aligns better with the distribution assumptions of these statistical methods.

**Figure 4:**
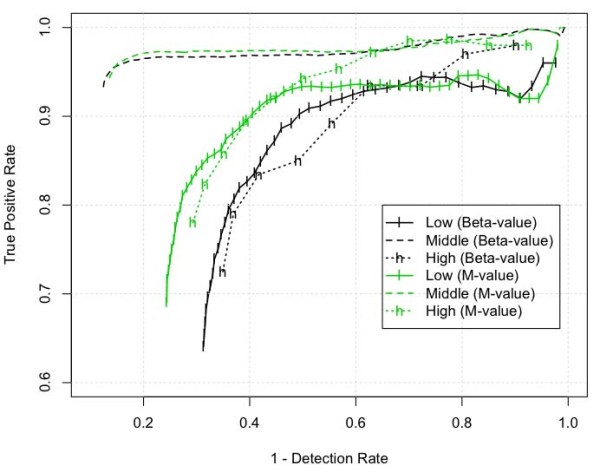
**Performance comparisons of Beta- and M-value in the range of low, middle and high methylation levels based on the relationship of 1 - Detection Rate versus True Positive Rate**.

Following a similar logical framework, we first used a simple t-test to compare two technical replicates of Sample A and two technical replicates of Sample B, and require a differentially methylated CpG site to have p-value < 0.05. We then separated these filtered CpG sites into the three analysis groups listed in the "Evaluation Dataset" subsection: low (2221 CpG sites for Beta-value; 2794 CpG sites for M-value), middle (6855 CpG sites for Beta-value; 6179 CpG sites for M-value) and high (457 CpG sites for Beta-value; 625 CpG sites for M-value) methylation analysis groups. In each analysis group, we sorted the CpG sites in decreasing order based on their absolute methylation difference between Sample A and B, i.e., $\left|\bar{Methylation_{A,i}}-\bar{Methylation_{B,i}}\right|$, where $\bar{Methylation_{A,i}}$ represents the average methylation level of Sample A at i^th ^CpG site. We then evaluate the performance of each method by selecting the top *N *CpG sites as an evaluation set, with *N *starting at 50 and incremented in steps of 50 until all sites were included in the evaluation set. For each evaluation set (top N CpG-sites), we calculated the True Positive Rate (TPR), where TPR was defined as the percentage of identified differentially methylated CpG sites being included in the True Positives (TP) set, i.e., *TPR *= |*TP*∩*CpG*_detected_|/|*CpG*_detected_|, where *CpG*_detected _represents the CpG sites included in the evaluation set. We also calculated the Detection Rate (DR) for each evaluation set, where DR was defined as the percentage of detected TP CpG sites among all TP CpG sites, i.e., *DR *= |*TP*∩*CpG*_detected_|/|*TP*|. Figure 4 shows the performance curves of Beta and M-value based on the relationship of 1 - DR versus TPR. The definition of these curves is similar with the ROC (Receiver Operating Characteristic) curve. In an ideal situation, the best performance point is located at the left top corner in the figure, where both DR and TPR are equal to 1. Comparing the performance curve of Beta and M-value, we can see that the M-value statistic performs much better than Beta-value in the low and high methylation range. In the middle range, their performance is similar although the Beta-value has slightly higher DR while the M-value has better TPR.

### Refinement of the basic differential methylation analysis

Similar to other hybridization techniques, there is an inherent level of variability associated with sample preparation, sample loading, the microarrays and the detectors. To address this variability it is very common to add a "minimum difference threshold" to select out CpG sites with little difference between two biological conditions. Next we want to evaluate the performance of the Beta-value and M-value statistics if we include a minimum difference threshold in addition to the p-value requirement.

**Figure 5:**
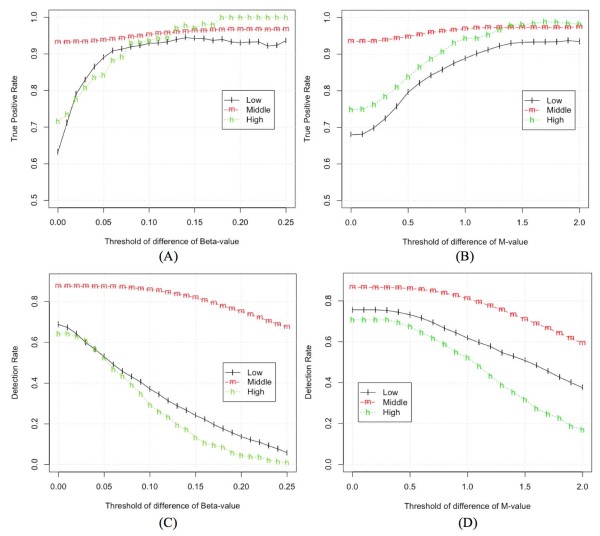
**Performance comparisons of Beta and M-value based on the True Positive Rate (TPR) and Detection Rate (DR) at different thresholds of methylation difference**. (A) TPR versus threshold of difference of Beta-value; (B) TPR versus threshold of difference of M-value; (C) DR versus threshold of difference of Beta-value; (D) DR versus threshold of difference of M-value.

After imposing a difference threshold, the identified differentially methylated CpG sites will have p-values < 0.05 and have the mean methylation level difference between A and B samples larger than the difference threshold. Figure 5 plots TPR and DR against the methylation difference threshold for the Beta-value and M-value methods. In Figure 5, at the starting point (with thresholds of difference equal 0), there are 9533 and 9535 identified CpG sites across the entire methylation range for Beta and M-value, respectively. At the end point (with thresholds of difference equal 0.25 and 2.0 for Beta and M-value, respectively), there are 5231 and 5168 identified CpG sites for Beta and M-value, respectively. This indicates that the threshold ranges for Beta and M-value in Figure 5 are comparable. Figure 5 shows that TPR improves as the difference threshold increases but the DR decreases. The performance of Beta-value and M-value methods is very similar for the middle analysis group (covering the approximate linear range of logit transformation). However, the performance of these methods differs substantially for the nonlinear (high and low) analysis groups. For the Beta-value statistic, the TPR increases as the difference threshold increases but DR drops dramatically. For the M-value statistic, the TPR increases more slowly, but DR remains high for much larger difference thresholds.

Figure 5 also provides some guidance for selecting the difference thresholds of Beta-value and M-value statistics. An ideal difference threshold would have both high TPR and high DR, but there is a tradeoff in selecting the threshold. From Figure 5, we can see that the TPR gradually increases with the difference threshold before stabilizing. Based on this, the difference threshold at the turning point of TPR can be set as the up-limit threshold because further increase of threshold will not improve TPR very much. On the other hand, the DR is almost constant at low thresholds and then gradually decreases with the increasing of difference threshold. So the difference threshold at the turning point of DR can be set as the down-limit threshold because it can increase the TPR without deteriorate the DR when DR is stabilized. Based on these guidelines, we suggest the range of threshold of M-value method should be about between 0.4 and 1.4 (or from 1.32 to 2.64 if we convert M-value to the non-log scale). For the Beta-value method, because of its severe *heteroscedasticity *in the low and high analysis groups, it is infeasible to provide a fixed threshold. We can only suggest the threshold of Beta-value for the middle analysis group, which is about between 0.05 and 0.15. It should be noted that these threshold ranges are dependent on the distribution of intensities in the dataset so ideally these thresholds should be determined for each dataset.

## Discussion

The Beta-value method has already been widely used to calculate methylation levels, and it is the manufacturer recommended method for analyzing Illumina Infinium HumanMethylation27 BeadChip microarrays. The M-value method has been widely used in the expression microarray analysis, and has been used to calculate methylation levels in some methylation microarray analyses [13]. However, to date there has been no systematic evaluation of the relationship between the Beta-value and M-value methods. In this study, we demonstrate that the two methods are related by a Logit transformation. They have an approximately linear relationship in the middle methylation range (defined as 0.2 to 0.8 for the Beta-value method) with a significant compression above and below this range for the Beta-value method. The Beta-value range is from 0 and 1 and can be interpreted as an approximation of the percentage of methlyation. However, because the Beta-value has a bounded range, this statistic violates the Gaussian distribution assumption used by many statistical methods, including the very prevalent t-test. In comparison, M-value statistic can be appropriately analyzed with these methods.

To compare the performance of Beta and M-value methods in identifying the differentially methylated CpG sites, we designed a methylation titration experiment. As we do not know the 'true' methylated CpG sites, we have defined a set of True Positives (TPs) based on high levels of correlation between the methylation and titration profiles. It is important to note that some true differentially methylated CpG sites may not be included in this set of TPs; at the same time, some false positives may also be included in the TPs. Fortunately, athough a small number of false positives or false negatives will affect the estimation of TPRs and DRs, but does not affect the overall performance comparisons between two methods (We did simulations by randomly adding or removing 10% TPs, and found the performance difference between Beta and M-values are consistent with the curves shown in Figure 4. The results were not included in the paper.). Comparing the performance based on top ranked CpG sites (ranked based on the absolute difference between two comparing groups), the M-value method has better detection power and a higher True Positive Rate (TPR) in the low and high methylation ranges due to its reduced *heteroscedasticity *in these ranges. In the middle methylation range, the Beta-value method has slightly better detection power than the M-value method but a decreased TPR.

In microarray differential analysis, adding a difference (or fold-change) threshold is another common practice and effective way to improve the TPR. However, due to the severe *heteroscedasticity *of the Beta-value method outside the middle methylation ranges, it is impossible to impose a constant difference threshold across entire methylation range for the Beta-value method. If a constant difference threshold is used for the Beta-value method, then the detection rate outside the middle methylation range is severely deteriorated. To solve this problem, Illumina proposed a customized model to detect differentially methylated CpG sites [21]. Basically, the model fits a parabola to the standard deviation as a function of Beta-value. However, this is inconvenient to implement, and the fitted parameters suggested by Illumina may change across different experiments under different conditions. Performing the same set of analyses using the M-value method demonstrates that using a constant difference threshold is appropriate and far easier to implement. Based on the comparison graphed in Figure 5 we suggest setting a threshold for the M-value method between 0.4 and 1.4 (or from 1.32 to 2.64 in the non-log scale).

## Conclusions

The Beta-value method has a direct biological interpretation - it corresponds roughly to the percentage of a site that is methylated. This makes the Beta-value very attractive when modeling the underlying biological effect. However, this interpretation is an approximation [22], especially when the data has not been properly preprocessed and normalized. From an analytical and statistical standpoint, the Beta-value method has severe *heteroscedasticity *outside the middle methylation range, which imposes serious challenges in applying many statistic models. In comparison, the M-value method is more statistically valid in differential and other statistic analysis as it is approximately *homoscedastic*. Although the M-value statistic does not have an intuitive biological meaning, it is possible to provide an accurate estimation of methylation status by modeling the distribution of the M-value statistic. In differential methylation analysis, we recommend using M-value because we can directly apply most statistical analysis methods designed for expression microarrays and it is easy to implement a difference threshold adjustment to improve the TPR. And the difference of M-value can be interpreted as the fold-change in the non-log scale. Although both Beta-value and M-value methods have some limitations, the two statistics are inter-convertible using Equation 3, enabling the use of the most appropriate method. We recommend using the M-value method for differential methylation analysis and also including the Beta-value statistic in final reports due to its intuitive biological interpretation.

## Methods

### Titration Samples

Similar to the titration design using Goldengate methylation chips by Bibikova and et al [12], we selected two samples with known methylation differences. Sample A is NA 10923 from Coriell Institute for Medical Research. It is a B-Lymphocyte sample from a male donor. Sample B is HTB-38 cell line from ATCC (http://www.atcc.org). It is a colon cancer sample from a female donor. Sample A and B were normalized into the same concentration, and then mixed in five different titration ratios. Table 1 shows the detailed information. The numbers in the row 2 and 3 in Table 1 are the percentage of sample A and B in the titration sample. Row 4 is the number of replicates of each sample.

### DNA Methylation Profiling using Illumina Infinium BeadChip Microarrays

The DNA samples were prepared following the guidelines suggested by the manufacturer (Illumina, Inc.), and then measured by Illumina Infinium HumanMethylation27 BeadChip, which measures 27578 CpG sites. The HumanMethylation27 BeadChip contains a pair of methylated and unmethylated probes designed for each CpG site. All experiments were conducted following the manufacturer's protocols by the Genomics Core at Northwestern University. The Illumina BeadChips were scanned with an Illumina BeadArray Reader and then preprocessed by the Illumina GenomeStudio software. Raw data have been deposited in the NCBI GEO database under the accession number of GSE23789.

We used the Bioconductor *methylumi *package [23] to input the methylation files outputted by Illumina GenomeStudio software and processed the methylation data using Bioconductor *lumi *package [24]. The methylation data was first passed QC and color balance check, and then background corrected and scaled based on the mean of all probes (using methylation simple scaling normalization (SSN) implemented in the *lumi *package). Beta-value and M-value statistics were calculated based on Equation 1 and 2. The related preprocessing functions are included in the Bioconductor *lumi *package (version > 2.0) [24]. As a prefiltering step, 82 CpG sites with more than 50% of samples having detection p-values worse than 0.0001 were filtered before the analysis. The Pearson correlation method was used to calculate the correlation between the titration and methylation profiles. Welch's t-test was used to identify the differentially methylated CpG sites.

## Authors' contributions

PD and SML initialized the idea of this paper. PD conducted all data analysis and drafted the manuscript. LH and SML supervised the methylation project. CH participated all discussions of data analysis and manuscript revisions. SML, PD, LH and WAK designed the titration experiment. XZ performed the titration experiment. All authors participated in the project at different stages, discussed the results and commented on the manuscript. All authors read and approved the final manuscript.
